# St. Louis Enhancing Engagement and Retention in HIV/AIDS Care (STEER): a participatory intersectional needs assessment for intervention and implementation planning

**DOI:** 10.21203/rs.3.rs-4225131/v1

**Published:** 2024-04-22

**Authors:** Debbie Humphries, Phillip Marotta, Yue Hu, Victor Wang, Greg Gross, Darius Rucker, Johnnie Jones, Faiad Alam, Tawnya Brown, Chelsey R. Carter, Donna Spiegelman

**Affiliations:** Yale University; Washington University In St Louis: Washington University in St Louis; Yale University; Yale University; Washington University In St Louis: Washington University in St Louis; Keys to Knowledge & Action Consulting; St. Louis Ryan White Planning Council; Yale University; Vivent Health; Yale University; Yale University

**Keywords:** Participatory Planning, Intersectional Needs Assessment, Intersectionality, Implementation Planning, Ending the HIV Epidemic

## Abstract

Background Missouri is one of seven priority states identified by the Ending the HIV Epidemic Initiative, and St. Louis contains almost half of the people living with HIV (PLWH) in Missouri. As St. Louis has a marked history of structural racism and economic inequities, we utilized the Intersectionality Based Policy Analysis (IBPA) framework to guide a participatory needs assessment for planning and program development. Methods The planning team included researchers, the lead implementer from our community partner, and two community representatives, and had biweekly 60–90 minute meetings for 18 months. The planning team discussed and approved all research materials, reviewed and interpreted results, and made decisions about outreach, recruitment, conduct of the needs assessment and development of the planned intervention. The needs assessment integrated information from existing data, (1) interviews with (a) PLWH (n=12), (b) community leaders (n=5), (c) clinical leaders (n=4), and (d) community health workers (CHWs) (n=3) and (e) CHW supervisors (n=3) who participated in a Boston University-led demonstration project on CHWs in the context of HIV and (2) focus groups (2 FG, 12 participants) with front line health workers such as peer specialists, health coaches and outreach workers. A rapid qualitative analysis approach was used for all interviews and focus groups. Results The IBPA was used to guide team discussions of team values, definition and framing of the problem, questions and topics in the key informant interviews, and implementation strategies. Applying the IBPA framework contributed to a focus on intersectional drivers of inequities in HIV services. The effective management of HIV faces significant challenges from high provider turnover, insufficient integration of CHWs into care teams, and organizational limitations in tailoring treatment plans. Increasing use of CHWs for HIV treatment and prevention also faces challenges. People living with HIV (PLWH) encounter multiple barriers such as stigma, lack of social support, co-morbidities, medication side effects and difficulties in meeting basic needs. Conclusions Addressing intersectional drivers of health inequities may require multi-level, structural approaches. We see the IBPA as a valuable tool for participatory planning while integrating community engagement principles in program and implementation design for improving HIV outcomes.

## BACKGROUND

Ending the HIV epidemic is a critical priority for global public health.(1–3) Despite progress in the prevention and treatment of HIV over the past decades, new infections occur daily in the United States and are highest among Black men, particularly those who have sex with men.(4) A significant number of people living with HIV (PLWH) in the United States are not receiving adequate care, with an estimated 21% being unaware of their HIV status, 25% not linked to care within 6–12 months of diagnosis, 50% not engaged in routine care/follow-up, and 25% not receiving antiretroviral therapy (ART).(4) Inequities in access to HIV treatment persist among young adults, people of color, and the lesbian, gay, bisexual, transgender and queer (LGBTQ) communities, highlighting the importance of an intersectional lens in developing and improving interventions to mitigate the complex web of inequities.(5–7)

To tackle these challenges, the Ending the HIV Epidemic Initiative (EHE) was launched in 2019 with the goal of a 90% reduction in new HIV infections in the U.S. by 2030.(1) The EHE prioritizes early diagnosis, rapid and effective treatment, prevention strategies such as pre-exposure prophylaxis, and quick response to potential outbreaks to make HIV a rare infection. Missouri is one of seven priority states under the EHE initiative due to higher rates of rural infections and presents unique challenges with a history of socioeconomic and racial inequities. In St. Louis black men and women are three to eight times more likely to be diagnosed with HIV than their white counterparts,(8–11) and high rates of HIV infection and lower engagement along the continuum of care are seen among young adults, people of color, and LGBTQ communities.(5–7) There are notable gaps in understanding the complex and overlapping dynamics that influence engagement and retention in HIV/AIDS care. The lack of an intersectional lens has obscured the structural vulnerabilities created by systemic responses to race, gender, sexual identities, and socioeconomic status, hindering comprehensive solutions for diverse communities.(9–11) Applying an intersectional lens to understand social and structural determinants of health through a participatory community approach can inform the adaptation and implementation of targeted interventions, such as Community Health Workers (CHWs) to address the complex and interconnected factors contributing to HIV inequities.

### Intersectionality-based policy analysis (IBPA) conceptual framework

**Figure 1:**
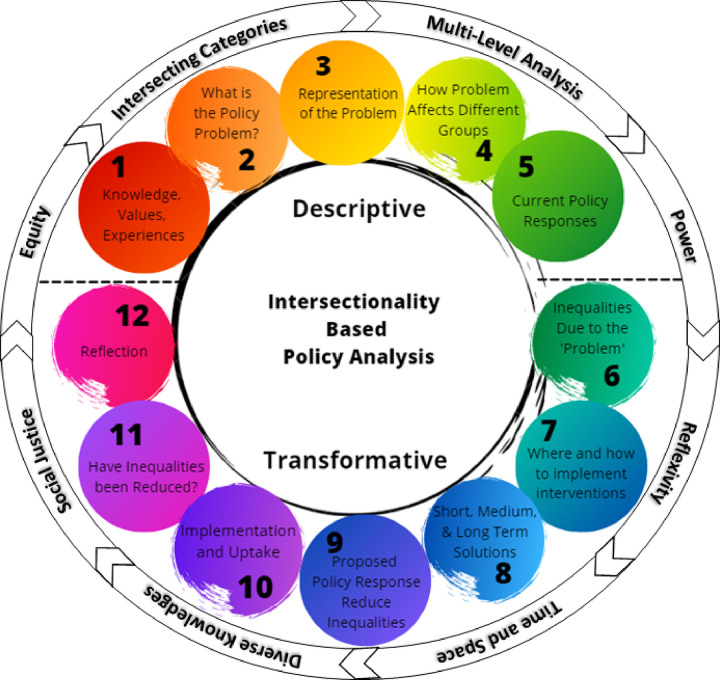
Intersectionality-Based Policy Analysis Framework. The descriptive and transformative questions are shown surrounded by the guiding principles to highlight the complex interaction of the two key components of the framework, and the importance of addressing both questions and guiding principles simultaneously. [Intersectionality-Based Policy Analysis Framework© 2021 by Debbie Humphries, Michelle Sodipo, Skyler Jackson; based on ideas of Olivia Hankivsky is licensed under Attribution 4.0 International.39]

We used the intersectionality-based policy analysis (IBPA) framework(12) to adapt a multilevel intervention and develop implementation strategies for a CHW-centered program to reduce inequities along the HIV care cascade. Intersectionality theory focuses on moving beyond examining individual factors, such as biology, socioeconomic status, sex, gender, and race, and elucidates the relationships and interactions among multiple interlocking systems across all levels of society for an individual or group.(13–15) The IBPA framework (Fig. 1) employs principles of social justice, power, and diversity of knowledge to interrogate overlapping systems and structures that affect policy and programmatic issues. (16) IBPA has been applied in various case studies and settings, including maternity care, HIV prevention strategies for gay men, and the criminalization of HIV nondisclosure in Canada, generating equity-focused perspectives that incorporate diverse viewpoints in both defining problems and seeking sustainable solutions (12, 17–19). The IBPA framework has also been used to identify intersectional drivers of inequities in access to HIV treatment services.(16) We selected this framework to guide intervention adaptation and implementation planning based on the explicit emphasis on discussing values and creating a participatory and equity-based space for leading the project, as well as an emphasis on praxis – using an intersectional lens to change systems.(20–22) Recent studies have demonstrated the value of the IBPA framework,(22–24) exemplified by its application to the U.S. COVID-19 policy response;(20) which underscores the growing significance of considerations of intersectionality in public health and highlights the transformative shift from analysis to actionable strategies.

The deployment of Community Health Workers (CHWs) to provide enhanced client support and strengthen client trust has been shown to be an effective strategy for enhancing client engagement,(25) providing acute treatment(26) and overcoming social and structural barriers that burden hard-to-reach populations.(27) CHWs are trusted members of the local community who deliver critical health promotion interventions, characterized by racial, socioeconomic and ethnic backgrounds, as well as lived experiences, that align with those of their client population.(28–31) This strategy has improved health outcomes for chronic conditions such as diabetes(32) and has been a valuable tool in responses to the COVID-19 pandemic.(33) In addition, CHWs have demonstrated a remarkable capacity to bridge gaps in the provision of HIV treatment as PLWH working with trained CHWs have higher rates of engagement along the treatment cascade and better health outcomes.(34–37) Enhanced personal contact between lay health workers and PLWH strengthens continuity of care for HIV clients by developing closer bonds with healthcare providers and reducing distrust of the medical system.(38) Integrating CHW roles into HIV treatment settings increases engagement in HIV treatment and care, although more implementation research is needed to understand the best ways to implement and scale up integration of CHWs into medical systems of care for PLWH.(34, 39)

### Participatory Planning and Needs Assessment

Participatory approaches to intervention adaptation and implementation increase community buy-in and enhance the viability, effectiveness and sustainability of interventions.(40–43) Participatory planning involves active participation and input from healthcare providers, community members, and other stakeholders to increase the likelihood that systems, service delivery and implementation are tailored to the community, leading to better health outcomes and improved quality of life.(44–48) Participatory planning is critical for offering patient-centered HIV care that is responsive to the unique needs and preferences of the community.(49, 50) By including PLWH in the process of adapting programs and implementation planning, teams can formulate more effective implementation strategies to strengthen the ability of CHWs and their organizations to reduce stigma and discrimination, increase adherence to treatment, and enhance the ownership by PLWH for their own care.(49, 51) Participatory planning can help identify and address barriers and facilitators to care, such as lack of access to transportation, concerns about confidentiality and levels of client-provider trust.(44) By integrating an intersectional approach with participatory planning in conducting a needs assessment, we enhanced our ability to accurately capture and respond to the community’s needs. The inclusion of participatory approaches not only acknowledges the unique insights and experiences of those directly affected by HIV but also cultivates a sense of investment and collaboration within communities.

Intervention Mapping and Implementation Mapping (IntMap and ImpMap)(52) provide a process for systematically developing an intervention and an implementation plan, beginning with a needs assessment.(53–57) The St. Louis Enhancing Engagement and Retention in HIV/AIDS Care (STEER) project conducted a participatory intersectional needs assessment to adapt a CHW intervention to enhance management of HIV/AIDS care and prioritize implementation strategies. We describe here a novel application of the IBPA within a participatory needs assessment that integrated community leaders into the research team to lay the groundwork for adapting and implementing a CHW-centered multi-level intervention to address the needs of PLWH in St. Louis.

## METHODS

### Setting

The study took place in St. Louis, Missouri (9.2021 through 5.2023), which has a high burden of HIV among historically marginalized communities. St. Louis residents comprised almost half (6,320 of the 13,109) of persons living with HIV in Missouri in 2018. In 2020, the population of St. Louis (300,576 people) was 46.4% Black, 46.5% White, 4.0% Hispanic or Latino, 0.3% Native American, and 3.4% Asian. (8) Approximately one in five people in St. Louis live below the poverty line.

### Research Aims and Design

The project utilized intervention mapping (IntMap) and implementation mapping (ImpMap)(52, 58) to adapt a CHW intervention and plan for its implementation. We report here on the initial phase, a participatory intersectional needs assessment utilizing the IBPA and development of a logic model of the problem of HIV care management and engagement.

## Research Process

Building the research team. The research team developed organically and intentionally by connecting with potential partners in St. Louis through existing networks of trust. Two academic partners and a community-based organization (CBO) leader with decades of experience in the St. Louis HIV treatment and prevention community jointly agreed to investigate how best to strengthen the implementation of CHW services in the context of HIV treatment. The CBO leader identified two trusted and innovative leaders in the PLWH community, and both met first with one of the academic leads to discuss the research plan, including the IBPA framework and participatory planning approach, and to address any questions. Both chose to join the bi-weekly planning team meetings as consultants.

The nested team structure included a planning team consisting of CBO leadership, community leaders, and university-based faculty and research assistants who met bi-weekly to develop research materials, review progress, address ongoing needs, and guide decision-making. The full research team, including experts in community health worker health program design, geographic modeling, and qualitative and quantitative research, were consulted to help synthesize insights from the research and plan the implementation. Local leaders in the St. Louis response to HIV on the St. Louis Fast Track Cities (FTC-STL) Steering Committee also provided a strong community perspective during the project. The team shared initial findings with the FTC-STL Steering committee to gather feedback.

Applying the IBPA. There are two primary components of the IBPA: guiding principles and questions.(12, 17) Eight guiding principles are provided to consider and integrate into the analytical process, along with 12 analysis questions. The first set of questions (Q1–5) are descriptive and focus on the team, the problem to be addressed, how the problem is framed, the impacts of the problem, and current policy responses. The subsequent questions (Q6–12) are transformative, addressing inequities in current impacts of the problem, potential solutions, ensuring implementation, metrics of success, and the team’s reflections on the use of the IBPA. Definitions of guiding principles were adapted from Humphries et al., (20) and the planning team identified examples of how each principle was applied in the planning and analysis process (Table 2). Draft answers to the IBPA analysis questions were developed by members of the planning team, and draft answers were presented to the full planning team for discussion and eventual approval.

Planning Team Activities. Early in the timeline (9.2021–5.2023) the planning team devoted a session to each planning team member sharing their knowledge, values, and experiences that contributed to their interest in this project (IBPA Q1: What knowledge, values, and experiences do you bring to the area of analysis?). Results were synthesized, reviewed, and approved by the planning team. Other activities included reviewing timelines, draft materials (such as IBPA responses, interview guides, recruiting materials and potential participants, analysis results, problem logic model), providing feedback on results, providing insights into community history and current community activities, as well as strategies to strengthen the implementation of CHWs in the HIV care system in St. Louis. IBPA principles were involved at each step to ensure that intersectional principles of power, structure, and social justice were incorporated throughout.

Data Collection and analyses. We conducted virtual interviews and focus groups via Zoom to identify barriers and facilitators to HIV care, as well as challenges and opportunities for integrating CHWs in the response to HIV in St. Louis. The planning team was responsible for interview materials and participant identification. St. Louis participants were identified through professional networks and personal contacts of the planning team to represent different clinical contexts and expertise. We interviewed CHWs and CHW supervisors who participated in a Boston University-led CHW HIV demonstration project (n = 6),(39, 46, 59) clinicians (n = 4), community leaders (n = 5), and people living with HIV (n = 12). We also conducted two focus groups (12 participants) with front line staff working in the field of HIV, such as peer specialists, health coaches and outreach workers. Demographic information for interview and focus group participants are included in Table 1. Interviewers and focus group moderators were inclusively trained, aiming to probe key points such as less socially accepted beliefs and perspectives. Analysis and coding were conducted via rapid qualitative analysis methods(60, 61) and mapped to the IBPA framework.(12, 17, 62) Interviews were transcribed either internally or by a professional transcription service, with transcripts checked by the interviewers. Each interview or focus group was summarized in a pre-designed template specific to each interviewee category (Appendix A). Templates were completed by one team member based on the interview transcript and then reviewed by the interviewer. The results within participant categories were compiled into separate matrices and summarized within the participant categories (A blank matrix is available as Appendix B). Two researchers developed and approved each summary, and all qualitative team members discussed and agreed on the final synthesized results. Once results were summarized for each participant group, a final matrix synthesizing results across interviewee categories was completed. Similar template categories across interviewee categories were aligned in the matrix, and those results were summarized. Draft findings and interview data were shared with the planning team, who reviewed conclusions, challenged interpretations, and helped to frame all final results.

Based on the interviews and focus groups, and oriented around the IBPA, a logic model of the problem of engagement and retention in HIV care was developed. Multiple drafts with different visualizations were developed before the planning team agreed on two frameworks that together embody the non-linearity and complexity of the problem.

We followed the STROBE reporting guidelines, and the checklist is attached as a supplemental file.

## Ethical Review

The study was reviewed and determined exempt by the Institutional Review Boards at Yale University and Washington University in St. Louis. Informed consent was obtained from all participants prior to data collection.

## RESULTS

Results of the intersectional needs assessment are presented through the application of the IBPA, the identified barriers to effective management of HIV, and the problem logic model.

### IBPA Values; Planning Team Values (Table 2, Table 3 Q1)

The guiding principles of IBPA include: recognizing the limitations of singular social categories, considering multi-level relationships in analyzing policy impacts, addressing power dynamics across various levels, practicing reflexivity for self-awareness, acknowledging the temporal and spatial dimensions of societal structures, valuing diverse sources and types knowledge, and striving for social justice and equity in policy formulation and analysis (see Table 2). Applications of these guiding principles in the needs assessment and intervention planning are detailed in Table 2. Each guiding principle manifested in multiple ways. For example, the team addressed the issue of power, including the awareness of differential power across care systems and within the planning team, by incorporating a structure for sharing leadership during planning team meetings. Part way through the project two planning team members proposed intentional inclusion of an open ‘unmeeting’[1] time during the regular biweekly meetings, to encourage all team members to raise questions, additional agenda items, thoughts, and broader systemic concerns. As the initiative progressed the unmeeting space evolved to ensure that everyone had a chance to participate and was eventually stewarded by a community leader and researcher together.

### IBPA Table (Table 3)

Our team emphasized values such as self-awareness, deep listening and reflection, and a commitment to actively engage in changing oneself and systems (Table 3 Q1: *What knowledge, values, and experiences do you bring to this area of analysis?*). In addressing Question 2 (Q2: *What is the ‘problem’ under consideration?*) we defined the problem as “structural oppression and systemic barriers create and perpetuate challenges for PLWH in their care management that then lead to inequities in HIV outcomes across intersectional categories.” The problem definition was extensively discussed in multiple planning team meetings until a consensus was reached. The team’s vision was to direct efforts towards systemic and structural changes to better support PLWH. In responding to Q3 we redefined the problem from the PLWH’s perspective, applying an equity lens to our analysis (Table 3 Q3: *How has our representation of the ‘problem’ come about?*). The response to Q4 highlighted how this problem representation differentially affects groups, with a focus on the structural and systemic issues faced by people with low income, people of color, and LGBTQ individuals, all of whom experience inequities in HIV outcomes (Table 3 Q4: *How are groups differentially affected by this representation of the ‘problem’?*). Q5 completes the descriptive part of the analysis by providing examples of current policy responses to HIV in the St. Louis area, such as the efforts of the Ryan White program, the FTSTL Steering Committee, and other CDC-funded programs for specific target populations (Table 3 Q5: *What are the current policy responses to the ‘problems’?*).

The second set of IBPA questions are transformative, bringing other lenses to the problem and encouraging a wider range of questions. We utilized existing publicly available data to identify current inequities, including higher rates of diagnosis and lower rates of care among Black gay and bisexual men, younger individuals, and people living below the poverty line (Q6: *What inequalities actually exist in relation to the ‘problem’?*). The planning team identified potential immediate interventions, such as providing resources to front line organizations to help PLWH meet basic needs and actively repurposing public spaces to be more welcoming and inclusive (Q7: *Where and how can (immediate) interventions be made to improve the problems in St. Louis?*).

The short-, medium- and long-term solutions identified drew upon published literature, the experiences and ideas of key informants, and the collective knowledge and experiences of the planning team. Short-term solutions focus on meeting basic human needs through social supports, deployment of CHWs, improved alignment of state and federal funds with needs, and increased distribution of support funds through front line organizations closely connected to communities with greater needs (Q8: *What are feasible short-, medium- and long-term solutions?*). Medium-term solutions include actively creating community spaces to process and heal historic trauma, increasing access to home testing, maintaining the social supports identified in immediate and short-term solutions, and integrating HIV services into primary care settings. These medium-term solutions aim to support individuals by making private and anonymous testing available and increasing the availability of routine care through standard health care channels. Long-term solutions involve addressing historic trauma, continuing biomedical research to develop a vaccine, and undertaking multipronged efforts to change the priorities and approaches of funding organizations, strengthening multidimensional measurement of health inequities, increasing representation of minorities in healthcare professions, and prioritizing work and funding through organizations that prioritize responding to impacts of race and racism more than emphasis on individual behavior change.

The next three questions address how the program will reduce inequities, ensure implementation and uptake, and measure changes in inequalities. We expect the program will most immediately reduce inequities by enhancing organizational capacity to integrate CHWs with lived experience from the local community, training them, innovating care team implementation, and promoting cooperation across multiple organizations (Q9: *How will proposed program reduce inequities?*). Regarding implementation and uptake, we have engaged with community leaders and groups through key informant interviews and other outreach activities to gather feedback, ideas and input on the intervention design and implementation strategies (Q10: *How will implementation and uptake be assured?*). Demonstrating that inequalities have been reduced will involve process measures (such as the availability of culturally appropriate medical information), outcome measures (such as reductions in inequities of HIV care engagement and viral suppression), and longer-term systemic change measures (such as reductions in stigma and medical mistrust) (Q11: *How will you know if inequalities have been reduced?*).

The final question of the IBPA reflects on the process, with planning team members noting that the IBPA framework facilitated dialogue and learning that shifted power dynamics and promoted social justice (Q12). Utilizing the IBPA led to deeper engagement and understanding of interlocking issues and identities, focused the conversation on structural and systemic issues within both the research process and the planned intervention (Q12: *How has the process of engaging in an intersectionality-based program analysis transformed: your thinking about relation and structures of power and inequity; the ways in which you and others engage in the work of policy development, implementation and evaluation; broader conceptualizations, relations and effects of power asymmetry in the everyday world*).

### Barriers to Effective Management of HIV for PLWH

**Figure 2:**
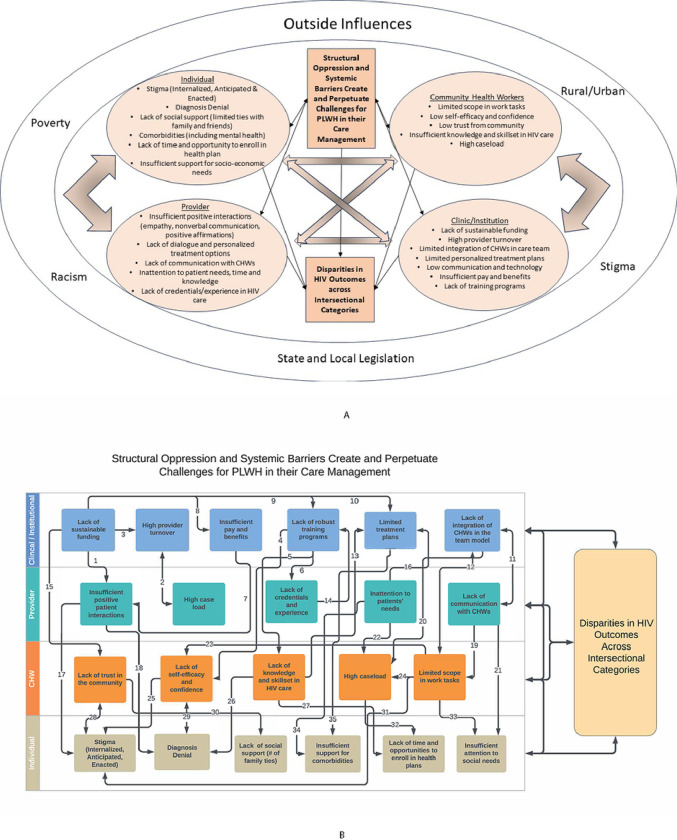
**a:** Nodal model showing systemic and contextual influences on problem **b:** Linear model of problem pathways based on relevant literature, interviews and focus groups

Across key informants, barriers to care for PLWH were identified at multiple levels, including the clinic/institutional level, provider level, CHW level and individual level (see Figure 2).

#### Clinic/Institutional Barriers.

High provider turnover, lack of sustainable funding for CHWs and other front line staff, insufficient pay and benefits for CHWs and front line staff, limited integration of CHWs into care teams, and limited organizational capacity for personalized treatment plans were all identified as barriers to effective management of HIV.

“[W]e saw a great turnover with the providers at this office, like if you look back over the last eight years there’s probably been 12 providers that have worked here, so people’s level of engagement often times was quite low and their follow through. So as clinicians picked up patients, they didn’t really have a good handle on the patient’s history, both from a social perspective or medical perspective… so I think if I put myself in a patient’s situation, I would have over time like decreased interest in my health care if I’m constantly experiencing clinician turnover. And I think because providers were here for such a short period of time they only touch on very superficial issues as they develop rapport with the patients, so like the deeper issues that affect their long-term care, I never really addressed.” [62–66] [Clinical Lead #2]

Adequate compensation for CHWs and similar front line staff, sustainability of funding, and availability of comprehensive training programs were noted as significant factors impacting organizational ability to provide effective assistance to PLWH. As one community leader noted,

“Most of our CHWs, and this is a nationwide thing, are supplied by grants, which means that their scope of service is then indicated by what that grant will allow, but also they don’t have long term viability for their career…. [What] I’ve learned is that the region, the state, and the nation value them, but it’s short lived when it comes to the operationality as well as the true infrastructure to support those workforces.” [130–133] [Community Lead #5]

#### Provider level Barriers.

Provider barriers that impact HIV management include challenges such as high workloads and limited time for providers to meet with each patient, as well as issues arising from provider knowledge and behaviors, such as limited awareness of CHWs and other front line staff. Clinical leaders reported being burdened with multiple responsibilities and having limited capacity to support clients in need.

I think most physicians that I work with… have a lot of other responsibilities, and they’ll see patients every two, three months. If people are doing less well, they might see him every three or four weeks. But I certainly think, and I have an expectation that if someone’s really struggling, a health coach is someone that could … contact the patient once or twice a week. I’ve had some health coaches that text patients every day to remind them to take their meds …This is something I certainly couldn’t do. [Clinical Lead #4]

In addition, clinical leaders noted challenges in understanding the responsibilities of new types of support staff as innovative roles are introduced to the team.

[O]ne of the problems with … Ryan White funded clinics, is that it provides money for a lot of type of roles like a health coach, but as soon as you get beyond the minimal office staff of your front desk person who handles taking in patients, and your medical assistants, the nurse, and the doctor, [nurse practitioner], you start adding people and you add people with more finely described roles. I think getting that information out so people understand who these people are and what they can offer is not easy…. [Clinical Lead #4]

#### CHW and Front line Staff Barriers.

Similar to clinical providers, CHWs and other front line staff encounter their own set of challenges that impact the care they provide to PLWH. These challenges include limited trust from the community, a lack of self-efficacy and confidence, insufficient knowledge and skills related to HIV, high caseloads, and scope of work limited by organizations.

“[T]he distrust and the disconnect between medical care is so vast, if you come with a prescriptive posture it’s over before it starts, you have to do something different.” [Clinical Lead #1]

Front-line health workers highlighted the importance of dedicating time to earn their clients’ trust.

“[Y]ou have to win their trust before you can test them [for HIV] because you have to … earn their trust, so they can open up and you can talk…. And when they trust you they can open up and they’ll talk to you, and you can have that one on one relationship where you can say hey let’s try this and then we talk in a week you let me know how you like that, and if you don’t like that then we’ll try something else.” [FG1-Prevention Specialist]

Front Line health workers also noted the importance of having time to discuss various approaches and the necessity of offering training and skill-building opportunities.

“I think we did more home visits back then, and we were able to spend a little more time with our clients doing some skill training and stuff like that. I think now we’re so focused on trying to get progress notes done that we’re not be able, you know we got so many clients that you can’t spend as much time with them as you used to.” [FG2-Supervisor]

#### PLWH Barriers.

Participants from all stakeholder groups identified challenges that impact the ability of PLWH to manage their HIV. Key themes included racial stigma, diagnosis denial, lack of social support, comorbidities, medication side effects, lack of time and opportunity to access health plans, and the inability to address basic needs.

“There’s always going to be a stigma attached to being HIV positive, and I think part of that is in the African American community, and I think the community health workers would have to ask that population why they drop out of care to actually get a real sense of why they’re not staying in care and take their meds, whether it be the other things that we’ve discussed already or if there’s some other reason why they’re not in care or staying in care.”] [Clinical Lead 3]

Addressing comorbidities such as mental health and chronic disease as well as challenges in meeting basic needs is crucial for supporting effective management of HIV by PLWH.

“We try to help people stay in care, but sometimes our clients have severe and persistent mental illnesses, they are not ready to be in care so sometimes they don’t follow up with services, it’s too regimented. So we kind of leave them on the fringe, but we still talk to them and try to say, well, when you need me call me… And so six months down the road they’re like … I need emergency housing, let me call [participant’s name].… So we get them back into care. (FG2-Supervisor)”

PLWH need access to a range of services, and may have multiple specialist care needs.

“[W]ith our clients and patients that we work with you know, besides going to their medical appointments, dental appointments, and the other specialist appointments they might have, they don’t have transportation, you know, they don’t feel well, you know they’re in a really difficult spot in their life and they feel alone.…” (FG1-Health Coach2)

### Logic Model of the Problem

Drawing on the results of the needs assessment and the HIV literature we developed two models of the problem (Figure 2), each highlighting different dimensions. The team decided both versions of the problem logic model were necessary to incorporate the complexity and external factors that influence the success or failure of future interventions. The first model (Figure 2a) adopts a nodal approach, emphasizing the mutually reinforcing challenges that affect management of HIV. The second model (Figure 2b) employs a linear approach, focusing on the pathways of influence between various barriers to HIV management.

In Figure 2a, the team positioned CHWs in a distinct node to draw attention to their role as intermediaries between institutions and individuals, while also highlighting their unique resource constraints. Although structural oppression and systemic barriers were specifically mentioned in the statement of the problem, Figure 2a also acknowledges that poverty, racism, stigma, legislation and rural/urban issues are important external influences.

In Figure 2b, the arrows illustrate the multiple pathways through which clinical and institutional challenges impact providers (arrows 1, 2, 6 and 11) and CHWs (arrows 4, 5, 12 and 15), as well as the pathways by which challenges at the provider and CHW level impact clients. Supporting documentation for the arrows in Figure 2b is provided in Appendix C.

## Discussion

This study conducted an innovative participatory intersectional needs assessment to facilitate the adaptation and implementation planning of an intervention integrating CHWs into the HIV care system. While prior applications of the IBPA have focused on policy change,(12, 63) this project represents the first use to our knowledge of the IBPA as a framework for conducting a participatory needs assessment. The IBPA fostered a focus on intersectional challenges, ensuring we paid explicit attention to less heard from voices. A recent meta-analysis on stigma and intersectionality further supports the approach, showing that intersectionality deepens the understanding of stigma experienced across social identities. (64)

During the IBPA process the research team encountered several challenges, including ensuring that both community and academic partners understood the IBPA and its role in informing the research. Balancing the need to complete the IBPA process with the priority of reviewing qualitative and quantitative documents in a timely manner required allocating purposeful time in meetings and creating working documents to track themes and discussions. Valuing the expertise while navigating the priorities of community partners without overburdening them, required careful planning.

Barriers to effective HIV management for PLWH, consistent with existing literature, were identified at the clinic/institutional, provider, CHW and client levels. Specific barriers reported at the clinic/institutional level included high turnover, insufficient funding for CHWs, limited integration of CHWs in care teams, and restricted organizational capacity for personalized treatment. Provider barriers reported encompassed high workloads, limited time, and inadequate awareness of CHWs skills. CHWs and front line staff reported facing challenges, including community distrust, low self-efficacy and confidence, insufficient HIV knowledge and skills, high caseloads, and a restricted scope of work. Client-level barriers reported included stigma, diagnosis denial, lack of social support, co-morbidities and medication side effects, healthcare access difficulties, and unmet basic needs. Previous studies have identified similar client-level barriers to HIV treatment engagement, including competing life activities, poor transportation, stigma, lack of insurance coverage, poverty, and beliefs about HIV care.(65–74) Among Black women living with HIV, HIV-related stigmas expressed by healthcare providers to them and poor quality HIV services have been identified as key barriers to treatment engagement.(65, 75, 76) At the organizational level, although research is limited, studies have identified challenges such as finding providers who speak the same language as patients and treat them with respect, providing team-based care, managing the cost of services, and navigating the logistics of the care system.(77, 78) Previously identified clinic and provider-level facilitators include patient friendly services and positive, trusting relationships with providers.(68, 79, 80)

The logic model of the problem employed two complementary formats: a nodal model and a linear pathways model, to capture the intersectional and systemic issues. While problem logic models typically offer a focused representation of a problem’s components, the planning team found it challenging to agree on a singular linear model. Utilizing two models allows for the incorporation of extensive chains of drivers and influences on HIV outcomes.

We report here on the initial steps of the IntMap and ImpMap protocols, with the needs assessment results intended to inform the future adaptation of the CHW approach. Results identifying implementation strategies and the development of an implementation plan to address identified barriers and enhance implementation outcomes(81–84) will be presented elsewhere.

## Limitations

This study was centered on St. Louis, and while its processes are relevant to other settings, specific barriers require validation in other populations. Due to challenges in recruiting younger PLWH and transgender individuals for interviews, future activities will include structured community conversations at St. Louis locations frequented by these groups to gather their input.

## Conclusions

Use of an intersectional framework contributed to identification of systemic and structural barriers to effective HIV management. The systemic and structural perspective was also apparent in the two complementary logic models of the problem developed by the team. Barriers to effective HIV management were identified across multiple levels, including organizations, providers, CHWs, front line staff and clients. Emphasizing an intersectional approach highlighted similar barriers as previous research to HIV management at the individual, provider and front line staff levels. At the clinic and institutional level additional barriers were identified such as high staff turnover, insufficient CHWs and insufficient CHW integration. This participatory intersectional needs assessment has identified local challenges and priorities to be addressed in the adapted intervention and implementation strategies.

## Abbreviations

HIV: Human Immunodeficiency Virus
PLWH: People Living with HIV
IBPA: Intersectionality Based Policy/Program Analysis
CHW: Community Health Worker
LGBTQ: Lesbian, Gay, Bisexual, Transgender and Queer
EHE: Ending the HIV Epidemic
IntMap: Intervention Mapping
ImpMap: Implementation Mapping
STEER: St. Louis Enhancing Engagement and Retention in HIV CARE
CBO: Community Based Organization
FTSTL: Fast Track St. Louis
CDC: Centers for Disease Control and Prevention
